# Cardiovascular Phenotype of Elevated Blood Pressure Differs Markedly Between Young Males and Females: The Enigma Study

**DOI:** 10.1161/HYPERTENSIONAHA.118.11975

**Published:** 2018-10-15

**Authors:** Chiara Nardin, Kaisa M. Maki-Petaja, Karen L. Miles, Yasmin, Barry J. McDonnell, John R. Cockcroft, Ian B. Wilkinson, Carmel M. McEniery

**Affiliations:** 1 From the Department of Medicine-DIMED, University of Padova, Italy (C.N.); 2 Division of Experimental Medicine and Immunotherapeutics, University of Cambridge, United Kingdom (C.N., K.M.M.-P., K.L.M., Y., I.B.W., C.M.M.); 3 Cardiff School of Health Sciences, Cardiff Metropolitan University, United Kingdom (B.J.M., J.R.C.).

**Keywords:** cardiac output, hypertension, vascular resistance, vascular stiffness

## Abstract

Blood pressure (BP) in young adults predicts BP in later life. We aimed to identify metabolic, hemodynamic, and autonomic characteristics associated with raised BP in young adults and whether these differ between males and females. Three thousand one hundred forty-five healthy subjects, aged 18 to 40 years, were grouped according to sex and BP category following the recent reclassification of BP as part of American Heart Association/American College of Cardiology 2017 guidelines. All individuals undertook a lifestyle and medical history questionnaire and detailed metabolic, hemodynamic, and autonomic assessments. Stage 1 hypertension and normal BP were the most common BP phenotypes in males (29%) and females (68%), respectively. In both sexes, cardiac output was positively associated with increasing BP category (*P*<0.001 for both). Similar positive trends were observed for heart rate and stroke volume in males (*P*<0.001 for both) and heart rate in females (*P*<0.001). Unlike in males, peripheral vascular resistance, aortic pulse wave velocity, and augmentation index were significantly increased in hypertensive females (*P*<0.001 for all) compared with the other BP categories. Most heart rate variability indices decreased across the BP categories, particularly in males. In young adults, metabolic and hemodynamic abnormalities associated with hypertension are already present at the elevated BP stage and the overall phenotype differed markedly between sexes. Whereas a cardiac phenotype was associated with elevated BP and hypertension in males, a vascular phenotype, characterized by elevated peripheral vascular resistance, aortic pulse wave velocity, and augmentation index, was dominant in females.

## Abstract 2

Supplemental Digital Content is available in the text.

Hypertension represents one of the most important causes of premature death and disability worldwide, although much is still unknown about the underlying cause.^1^ Interestingly, data from the Framingham Heart Study^2^ demonstrate that the increased risk associated with high blood pressure (BP) is not simply confined to those individuals with hypertension but applies to those with high-normal BP as well. Indeed, in a large meta-analysis of ≈1 million adults aged 40 to 89 years, there is no evidence of a threshold, down to at least 115/75 mm Hg, for cardiovascular risk related to BP.^3^

The recent reclassification of BP as part of the American Heart Association/American College of Cardiology 2017 guidelines defines hypertension as systolic BP (SBP) ≥130 mm Hg and diastolic BP (DBP) ≥80 mm Hg. The guidelines also introduce a new arbitrary BP category called elevated BP (EBP), defined as SBP 120 to 129 mm Hg and DBP ≤80 mm Hg.^4^ However, the cardiovascular risk associated with EBP, as defined by the guidelines, is still largely unknown in younger subjects. BP tracks strongly throughout life,^5^ and small interindividual differences in BP at an early stage become increasingly magnified over time.^6^ Moreover, exposure to mild BP elevation during youth increases cardiovascular risk later in life, independently of BP.^7^ Although a number of studies have examined mechanisms and consequences of BP elevation in older adults, the seeds of future cardiovascular risk are likely to be set in youth, making it important to understand the mechanisms underlying early elevations in BP.

The aim of this study, therefore, was to examine metabolic, hemodynamic, and autonomic characteristics across a range of BP categories in a large cohort of healthy young adults, with limited exposure to cardiovascular risk factors. We hypothesized that the mechanisms associated with hypertension in young people are already evident at the elevated stage of BP, and we wished to determine whether these differed between males and females.

## Methods

The data that support the findings of this study are available from the corresponding author on reasonable request.

### Participants

The Enigma study is a long-term follow-up study of young individuals, investigating the natural history of BP with regard to clinical, physiological, and genetic characteristics.^8^ Individuals were selected at random from 2 University populations in the United Kingdom (Cambridge and Wales; response rate ≈70%). Detailed hemodynamic measurements were available in 3145 subjects, aged between 18 and 40 years (1564 males and 1581 females). Patients with diabetes mellitus and evidence of cardiovascular disease (CVD) and renal failure were excluded, as well as those with systemic inflammatory diseases. Subjects taking any vasoactive medication were also excluded. Approval for all studies was obtained from the Local Research Ethics Committees (Cambridge, UK, and Iechyd Morgannwg Health Authority, South Wales, UK), and written informed consent was obtained from each participant. All procedures were followed in accordance with institutional guidelines.

### Protocol

All subjects completed a detailed lifestyle and medical history questionnaire; height and weight were assessed, and BMI was calculated. After 15 minutes of seated rest, brachial BP and radial artery waveforms were recorded. After 20 minutes of supine rest, brachial BP and radial artery waveforms were reassessed, and aortic pulse wave velocity (aPWV), cardiac output (CO), and heart rate variability (HRV) were determined, as described below.

### Hemodynamics

Brachial BP was recorded in the dominant arm using a validated semiautomatic oscillometric sphygmomanometer and an appropriately sized cuff (HEM-705CP; Omron Corporation, Japan), with a study operator present (research nurse or assistant). Three readings were taken over a 5-minute period. A high-fidelity micromanometer (SPC-301; Millar Instruments) interfaced with a computer using SphygmoCor software (SphygmoCor; AtCor Medical, Australia) was used to record radial artery waveforms from the wrist of the dominant arm and generate a corresponding central (ascending aortic) waveform, as already validated.^9^ From this, central (aortic) BP, measures of arterial wave reflections (augmentation index [AIx] and augmentation pressure [AP]), mean arterial pressure (MAP), heart rate (HR), and pulse pressure amplification were obtained; aPWV was calculated from waveforms recorded at the carotid and femoral sites using the same device. All pressure waveforms were sampled over ≈30 s at each site and were recorded in duplicate or triplicate if results differed by >4% (AIx) or 0.5 m/s (aPWV) over repeated readings.^10^ CO, cardiac index, and stroke volume (SV) were assessed using a noninvasive, inert gas rebreathing technique (Innocor, Innovision A/S, Denmark)^11^ which has previously been validated against thermodilution and direct Fick methods.^12^ In brief, while resting, subjects continuously rebreathed a gas mixture (1% SF_6_, 5% N_2_O, and 94% O_2_) over 20 s, with a breathing rate of 15/min. Expired gases were sampled continuously and analyzed by an infra-red photoacoustic gas analyzer, for the determination of CO, SV, and cardiac index. Peripheral vascular resistance (PVR) was estimated using the formula: PVR (dynes s cm^5^)=MAP (mm Hg)×80/CO (L/min). All measurements were made by trained investigators. The within- and between-observer measurement reproducibility values for the arterial stiffness measurements were in agreement with our previously published data.^10^ The coefficient of variation of repeated determinations of CO was <10%.

### Heart Rate Variability

The SphygmoCor device (SphygmoCor; AtCor Medical, Australia) was used to provide HRV measurements. After 20 minutes of supine rest, a 3-lead ECG signal was recorded over 10 minutes at a sampling rate of 1024 Hz. The analysis of time-domain components of HRV was assessed using the mean and SD of inter-beat (RR) intervals (ms), as already validated.^13^ Frequency-domain components were then estimated by Fast Fourier Transform to calculate the powers in the high frequency (from 0.15 to 0.40 Hz) and low frequency (from 0.04 to 0.15 Hz) ranges, as described previously.^13^ High frequency and low frequency components of HRV were expressed in normalized units; the low frequency:high frequency ratio was also calculated.

### Biochemical Measurements

Blood samples were collected from the antecubital vein under fasting conditions. TC (total cholesterol), LDL-C (low-density lipoprotein cholesterol), HDL-C (high-density lipoprotein cholesterol), TG (triglycerides), and serum glucose and creatinine were assessed using standard laboratory methods.

### Statistical Analysis

Data were analyzed using SPSS software (version 25.0). Subjects were grouped according to seated brachial BP following the American Heart Association/American College of Cardiology 2017 guidelines for the classification of hypertension^4^: normal BP (NBP: SBP <120 mm Hg and DBP <80 mm Hg); EBP (SBP 120–129 mm Hg and DBP <80 mm Hg); hypertension stage 1 (HT1: SBP 130–139 mm Hg or DBP 80–89 mm Hg); and hypertension stage 2 (HT2: SBP ≥140 mm Hg or DBP ≥90 mm Hg). Data were analyzed separately for males and females and differences between BP groups were evaluated using 1-way ANOVA for continuous variables and χ^2^ test for categorical variables. Post hoc analyses were conducted using the Tukey method. ANCOVA was used to assess differences between BP groups in all hemodynamic parameters after adjusting for age and ethnicity. aPWV was adjusted for HR and MAP, whereas AIx and AP were adjusted for HR and height. All values represent means±SD, and a *P* value of <0.05 was considered significant.

## Results

### Demographic and Metabolic Characteristics

Demographic and metabolic characteristics are presented in Tables 1 and 2 for males and females, respectively. The proportion of the different ethnic groups is shown in Table S1 in the online-only Data Supplement. HT1 was the most common BP phenotype in males (29%), whereas NBP was the most common BP phenotype in females (68%), and the prevalence of EBP, HT1, and HT2 in males was more than twice that in females. A breakdown of specific BP phenotypes is provided in Table S2.

**Table 1.**
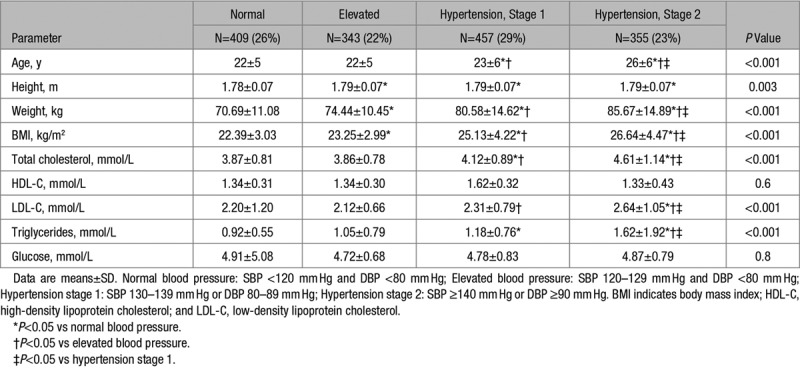
Demographic and Metabolic Characteristics in Males

**Table 2.**
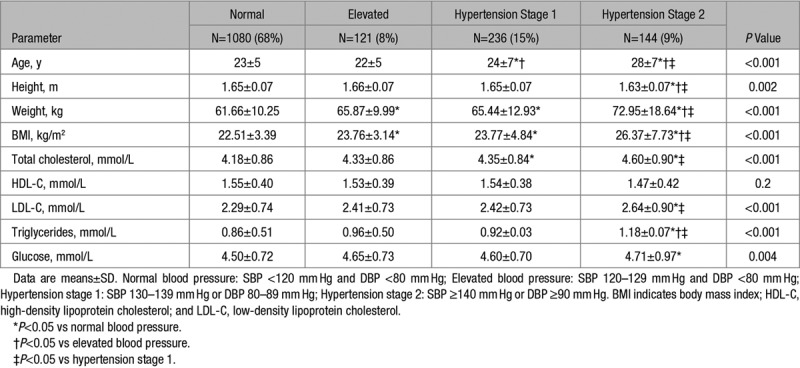
Demographic and Metabolic Characteristics in Females

For both males and females, there were significant, positive trends for higher age, weight, and BMI with increasing BP category (*P*<0.001 for all). There were also significant positive trends for TG with increasing BP category in males (*P*<0.001 for overall trend) and for TC, LDL-C (*P*<0.001 for both), and serum glucose (*P*=0.004) in females. Additional data about lifestyle factors and biochemistry are shown in Tables S3 and S4 for males and females, respectively.

### Hemodynamic Characteristics

**Figure 1.**
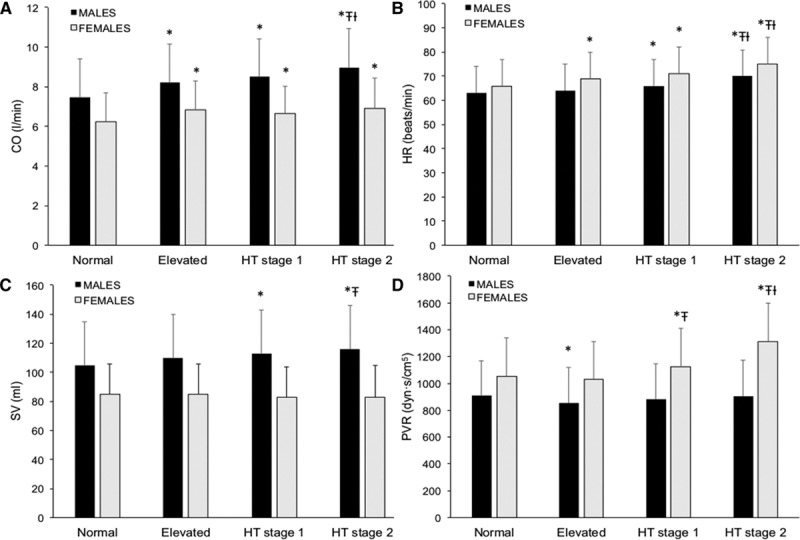
Hemodynamic parameters according to blood pressure (BP) category in males and females. **A**, Cardiac output (CO); (**B**) heart rate (HR); (**C**) stroke volume (SV); and (**D**) peripheral vascular resistance (PVR). *P<0.05 vs Normal. Ŧ*P*<0.05 vs elevated BP. ł*P*<0.05 vs hypertension (HT) stage 1.

**Figure 2.**
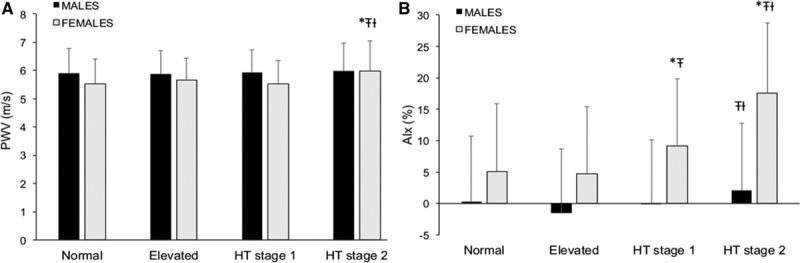
Hemodynamic parameters according to blood pressure (BP) category in males and females. **A**, Augmentation index (AIx) and (**B**) aortic pulse wave velocity (PWV). *P<0.05 vs Normal. Ŧ*P*<0.05 vs elevated BP. ł*P*<0.05 vs hypertension (HT) stage 1.

Detailed seated and supine hemodynamic characteristics are presented in Tables 3 and 4 and in Figures 1 and 2. In males, CO increased across the 4 BP categories (*P*<0.001 for overall trend), with a difference of 1.48 L/min between NBP and HT2. This trend was attenuated, but remained significant, after adjusting for body size (body surface area; *P*<0.001 for overall trend). Similar positive trends were observed for both HR and SV (*P*<0.001 for both), with differences of 7 beats per minute and 11 mL between NBP and HT2 for HR and SV, respectively, although the trend for SV was no longer significant after adjusting for body size (*P*=0.4). AP and AIx were highest in HT2 (*P*<0.001 for both); PVR was lowest in the EBP group (*P*=0.04), whereas aPWV did not differ between the BP categories (*P*=0.7) after adjustment for height and HR (AIx) or HR and MAP (aPWV). In females, CO was significantly elevated in the hypertensive categories compared with the normotensive group (*P*<0.001 for overall trend). This pattern remained after adjusting for body size (*P*<0.001 for overall trend). Interestingly, although HR increased across the 4 BP categories (*P*<0.001 for overall trend), with a difference of 9 beats per minute between the lowest and the highest category, unlike in males, there was no difference in SV between the 4 BP categories (*P*=0.4; difference of 2 mL between the highest and the lowest category). Moreover, adjusting for body size revealed a significant decline in SV with increasing BP category (*P*=0.002 for overall trend). Similar to males, AP and AIx were highest in HT2 (*P*<0.001 for both) after adjustment for height and HR. However, unlike males, PVR was also highest in HT2 (*P*<0.001 for overall trend), as was aPWV (*P*<0.001 for overall trend), after adjustment for HR and MAP.

**Table 3.**
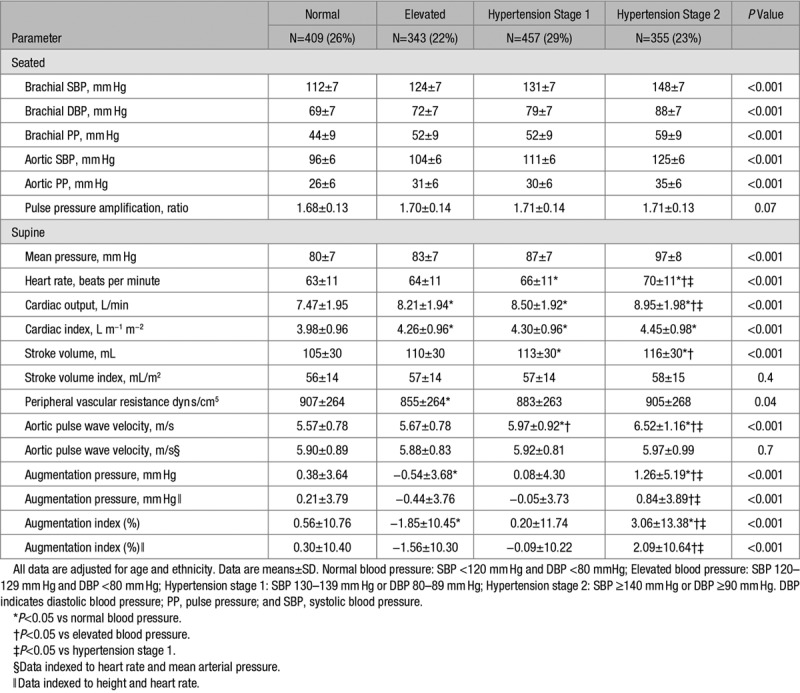
Seated Brachial and Aortic Blood Pressure Values and Supine Hemodynamic Characteristics in Males

### HRV Characteristics

HRV data were available in a subgroup of 961 subjects (465 males and 496 females) and are summarized in Tables S5 and S6, for males and females, respectively. In males, total power decreased significantly across the BP categories (*P*=0.02 for overall trend), as did the standard deviation of normal-to-normal intervals (SDNN; *P*=0.003 for overall trend), the proportion of successive NN intervals greater than 50 ms divided by the total number of NN intervals (pNN50; *P*=0.001 for overall trend), the root mean square of successive differences between RR intervals (RMSSDD; *P*=0.02 for overall trend), and triangular index (*P*=0.03 for overall trend). In females, SDNN and pNN50 were significantly reduced in HT2 compared with EBP and NBP groups (*P*=0.03 and *P*=0.007, respectively), and RMSSDD showed a significant general decreasing trend across the EBP and hypertensive categories without any specific differences between them (*P*=0.03).

**Table 4.**
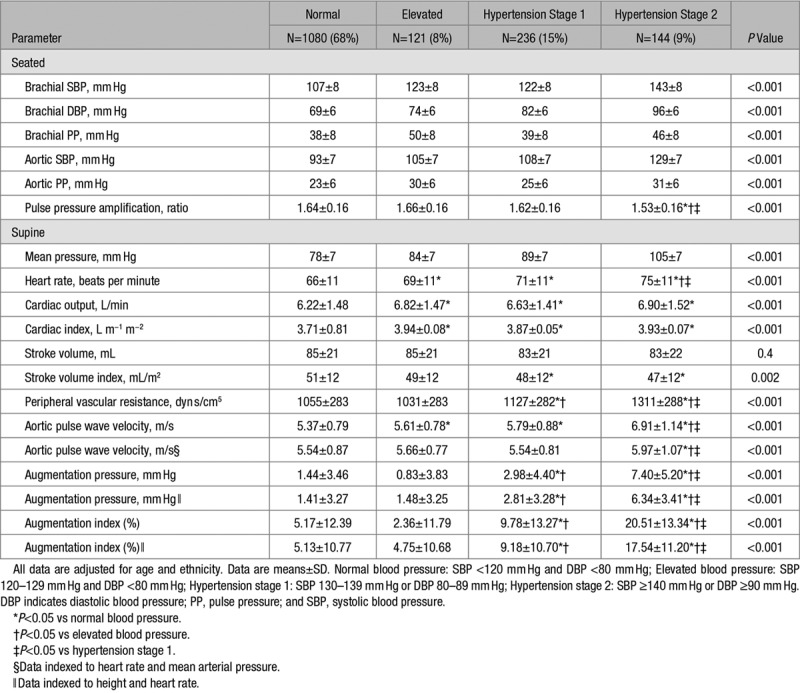
Seated Brachial and Aortic Blood Pressure Values and Supine Hemodynamic Characteristics in Females

## Discussion

Our major findings were that key cardiovascular phenotypes associated with hypertension in young adults were also present in individuals with EBP and differed markedly between males and females. Although elevated CO was common to both sexes, albeit driven by different mechanisms, hypertensive males displayed a predominantly cardiac phenotype with lower or normal PVR, whereas females displayed a predominantly vascular phenotype, relating to the resistance vasculature and larger arteries, and characterized by elevated PVR, AIx, and aPWV.

The higher prevalence of EBP, HT1, and HT2 in males than in females, observed in the current study, was not surprising as it is well established that BP is lower in females than in males from adolescence until the fifth decade, when the prevalence of hypertension in females increases steeply.^14–16^ Moreover, the high proportion of HT1 in males reflects the definition of hypertension (BP ≥130/80 mm Hg) used in the current study, following the American Heart Association/American College of Cardiology 2017 guidelines.^4^ Although the guidelines are controversial,^17^ in most cases, lifestyle modification rather than pharmacological treatment is still recommended.^18^

Relatively few studies have investigated the hemodynamic mechanisms underlying mild BP elevation in young adults, and they have mainly focused on prehypertensive individuals as defined by The Seventh Report of the Joint National Committee on Prevention, Detection, Evaluation, and Treatment of High Blood Pressure guidelines.^19^ Data from the Strong Heart Study^20^ reported an increase in CO and HR, together with increased left ventricular mass, associated with prehypertension and hypertension in a large cohort (N=1940) of young people aged 14 to 39 years. However, the high prevalence of diabetes mellitus and obesity in this population could have contributed to the adverse cardiovascular profile described in the prehypertension group. In the current study, we have considered healthy young adults with limited exposure to cardiovascular risk factors. Grouping subjects according to the American Heart Association/American College of Cardiology 2017 guidelines allowed us to compare cardiovascular characteristics across a range of BP levels, while gaining mechanistic insights to the impact of the new guidelines in young adults.

We observed that increased CO was associated with EBP and hypertension in both males and females, even after adjusting for body surface area. This means that the elevation of CO was not simply secondary to increased body size but could represent the predominant hemodynamic disturbance involved in the early elevation of BP in young adults. In addition, our findings suggest that the mechanisms underlying elevated CO in young people are influenced by sex. CO is the product of SV and HR, and although both variables increased across the BP groups in males, only HR showed a significant positive trend with increasing BP category in females. Indeed, SV adjusted for body surface area actually declined with increasing BP category in females, confirming the marginal role of SV in the elevation of CO in young females. Previous investigators have described the phenomenon of a hyperdynamic circulation in young males, preceding the development of sustained hypertension, characterized by normal PVR but increased SV and HR.^21^ In addition, our previous data from the Enigma study, which focused on the pathogenesis of isolated systolic hypertension, reported an increase in CO, SV, and aPWV in young participants (predominantly males) with isolated systolic hypertension compared with normotensives.^8^ A hyperdynamic, high CO phenotype was also described by Romero et al^22^ in their young patients with isolated diastolic/predominantly diastolic hypertension, although their cohort was very small (N=46). Nevertheless, their data suggest that a hyperkinetic circulation could be also involved in the pathogenesis of isolated diastolic hypertension/predominantly diastolic hypertension in young people. An elevated CO could represent the principal early hemodynamic disturbance in both young males and females with mild BP elevation and initiate a cascade of hemodynamic adaptations that differ by sex, although this hypothesis remains to be tested.

In the present study, PVR was lowest in subjects with EBP. This pattern could represent an initial compensatory lowering of PVR in response to the elevated CO, perhaps to protect end organs from potentially damaging increases in blood flow. Moreover, the normal PVR observed in hypertensive males may actually signify a failure of the peripheral vasculature to adapt appropriately to the high flow (CO). In contrast, the markedly increased PVR in hypertensive females suggests a predominant and, possibly, earlier involvement of PVR in the development of sustained hypertension in females. Interestingly, a similar trend was observed for aPWV which was associated with hypertension in females, but not in males. Moreover, the magnitude of differences in AP and AIx between those with NBP and the hypertensive categories were more marked in females than in males. Taken together, these data suggest that a vascular phenotype, characterized by increased PVR, increased wave reflections, and increased arterial stiffness, may dominate the development of sustained hypertension in females. In contrast, a more cardiac phenotype, characterized by an increase of both CO and SV, may dominate the development of sustained hypertension in males. These sex differences in hemodynamic phenotypes might explain the greater tendency of hypertensive females to develop end-organ damage. Indeed, data from the HARVEST study (Hypertension and Ambulatory Recording Venetia Study), focusing on end-organ damage in a young- to middle-aged cohort screened for HT1, demonstrated that microalbuminuria and left ventricular hypertrophy were more common in females than in males.^23^

HRV represents a widely used noninvasive tool to estimate cardiac autonomic activity.^13^ Previous data support the involvement of both sympathetic and parasympathetic nervous systems in the increased CO associated with early elevations of BP.^21,24–27^ In the current study, although there were no significant differences in components of HRV between NBP and EBP groups, most HRV indices decreased across the BP categories, particularly in males. However, further studies, adequately powered, are needed to investigate autonomic nervous system activity in young people with mild BP elevation.

There are several limitations of the current study. Its cross-sectional design does not permit us to examine causality or to distinguish parallel from sequential pathways involved in the development of sustained hypertension. Our stratification was based on BP measured on a single occasion, and we cannot exclude a possible white-coat effect among the young participants, despite the standardized measurement conditions. We did not investigate microvascular structure or function and so cannot determine the precise factors underlying the increased AIx and PVR in hypertensive females. Although there is some evidence suggesting an influence of the phase of menstrual cycle on arterial stiffness and wave reflections in females,^28,29^ we did not collect these data and so cannot assess this in the current study. Moreover, we included a small number of subjects with asthma and taking inhaled corticosteroids. Because asthma has been associated with hypertension,^30^ we cannot exclude a possible interference of asthma with BP values in the current data. Finally, our analyses based on HRV may have been underpowered to explore the involvement of sympathetic and parasympathetic activity in early elevations of BP in this cohort. The large cohort of young individuals and the long-term follow-up study design are strengths of the Enigma study, which should enable us to determine the causal mechanisms of hypertension in the future.

## Perspectives

BP in young adults predicts BP in later life, and individuals with EBP during adolescence or young adulthood are at greater risk of developing sustained hypertension and its pathological consequences. Therefore, understanding the mechanisms underlying early elevations in BP is important for appropriate intervention and follow-up of those individuals at high risk of developing sustained hypertension. Our data suggest that hemodynamic changes are incremental and not simply confined to a diagnosis of hypertension. As such, an increased CO could represent the common, initiating mechanism involved in the early elevation of BP. However, the predominantly cardiac phenotype of hypertension observed in males versus vascular phenotype of hypertension observed in females suggest that responses to pharmacotherapy will be heterogeneous between sexes and that targeting of therapy to underlying hemodynamic phenotypes could be a useful strategy to optimize BP control. As such, vascular phenotypes may benefit from peripheral vasodilators, whereas cardiac phenotypes may benefit from diuretics, if driven by volume overload, or β1 antagonists, if driven by cardiogenic mechanisms. Clearly, further trials are required before targeting therapy in this way can become the accepted approach to BP control in routine clinical practice.

## Appendix

The Enigma Study Investigators: Samantha Benedict, John Cockcroft, Zahid Dhakam, Lisa Day, Stacey Hickson, Kaisa Maki-Petaja, Barry McDonnell, Carmel McEniery, Jessica Middlemiss, Karen Miles, Maggie Munnery, Pawan Pusalkar, Christopher Retallick, Ramsey Sabit, James Sharman, Jane Smith, Jean Woodcock-Smith, Edna Thomas, Sharon Wallace, Ian Wilkinson, Yasmin.

## Acknowledgments

We acknowledge help from the Fondazione per la Ricerca Biomedica Cardiovascolare (FORIBICA) Foundation.

## Sources of Funding

Data collection for this work was funded by the British Heart Foundation (PG03/050/15366 and FS/06/005). This work was funded, in part, by the National Institute for Health Research (NIHR) Cambridge Biomedical Research Centre. The views expressed are those of the authors and not necessarily those of the NIHR.

## Disclosures

None.

## Supplementary Material

## Novelty and Significance

### What Is New?

- An increased cardiac output could represent the common, initiating mechanism involved in the early elevation of blood pressure.
- A predominantly cardiac phenotype was associated with the development of hypertension in males.
- A vascular phenotype, characterized by elevated peripheral vascular resistance, aortic pulse wave velocity, and augmentation index, was associated with the development of hypertension in females.

### What Is Relevant?

- Sex differences in hemodynamic phenotypes suggest that responses to pharmacotherapy will be heterogeneous and that targeting of therapy to underlying hemodynamic phenotypes could be a useful strategy to optimize blood pressure control.
- Further studies are required before targeting therapy in this way can become the accepted approach to blood pressure control in routine clinical practice.

### Summary

Cardiovascular phenotypes underlying hypertension in young adults are already present at the elevated blood pressure stage and differ markedly between males and females.
