# Supplementary material for: Cardiovascular Phenotype of Elevated Blood Pressure Differs Markedly Between Young Males and Females: The Enigma Study
Source: Hypertension. 2018 Oct 15;72(6):1277–84. doi: 10.1161/HYPERTENSIONAHA.118.11975 (PMC6221425; doi:10.1161/HYPERTENSIONAHA.118.11975)
Supplement: Supplementary file 1 [file hyp-72-1277-s001.docx]

**Data Supplement**

**The cardiovascular phenotype of elevated blood pressure differs markedly between young males and females: The Enigma Study**

Chiara Nardin^1,2^, Kaisa M Maki-Petaja^2^, Karen L Miles^2^, Yasmin^2^, Barry J McDonnell^3^,

John R Cockcroft^3^, Ian B Wilkinson^2^, Carmel M McEniery^2^

On behalf of The Enigma Study investigators

^1^Department of Medicine - DIMED

University of Padova

Padova, Italy

^2^Division of Experimental Medicine and Immunotherapeutics

University of Cambridge

Cambridge, UK

^3^Cardiff School of Health Sciences

Cardiff Metropolitan University

Cardiff, UK

**Correspondence:**

Dr Carmel McEniery

Division of Experimental Medicine and Immunotherapeutics

University of Cambridge, Addenbrooke’s Hospital Box 110, Cambridge, CB2 0QQ, UK.

Tel: +44 1223 217564; Fax: +44 1223 216893; Email: cmm41@cam.ac.uk

**Short Title:** Cardiovascular phenotypes in young males and females

| **Reference**  1. Levey AS, Stevens LA, Schmid CH, Zhang YL, Castro AF, 3rd, Feldman HI, Kusek JW, Eggers P, Van Lente F, Greene T, Coresh J, Ckd EPI. A new equation to estimate glomerular filtration rate. *Ann Intern Med*. 2009;**150**:604-12.  **Table S1. Number of subjects according to sex, BP phenotype and ethnic group.** | | | | | | | |
| --- | --- | --- | --- | --- | --- | --- | --- |
| ***MALES*** | | | | | | | |
| ***Category*** | ***No (%)*** | ***Ethnic group (%)*** | | | | |  |
|  |  | ***Caucasian*** | ***Asian*** | ***Far Eastern*** | ***Afro-Caribbean*** | ***Other*** |  |
| **NBP** | 409 (26) | 292 (71) | 31 (8) | 42 (10) | 27 (7) | 17 (4) |  |
| **EBP** | 343 (22) | 266 (78) | 13 (4) | 26 (8) | 25 (7) | 13 (3) |  |
| **HT1** | 457 (29) | 353 (77) | 24 (5) | 34 (8) | 28 (6) | 18 (4) |  |
| **HT2** | 355 (23) | 294 (83) | 18 (5) | 12 (3) | 21 (6) | 10 (3) |  |
| Total | 1564 | 1205 (77) | 86 (6) | 114 (7) | 101 (6) | 58 (4) |  |
| ***FEMALES*** | | | | | | | |
| ***Category*** | ***No (%)*** | ***Ethnic group (%)*** | | | | |  |
|  |  | ***Caucasian*** | ***Asian*** | ***Far Eastern*** | ***Afro-Caribbean*** | ***Other*** |  |
| **NBP** | 1080 (68) | 841 (78) | 58 (5) | 75 (7) | 75 (7) | 31 (3) |  |
| **EBP** | 121 (8) | 107 (88) | 3 (3) | 1 (1) | 4 (3) | 6 (5) |  |
| **HT1** | 236 (15) | 193 (82) | 9 (4) | 12 (5) | 13 (5) | 9 (4) |  |
| **HT2** | 144 (9) | 125 (87) | 7 (5) | 2 (1) | 9 (6) | 1 (1) |  |
| Total | 1581 | 1266 (80) | 77 (5) | 90 (6) | 101 (6) | 47 (3) |  |
| NBP= Normal blood pressure (SBP <120 mmHg and DBP < 80 mmHg); EBP= Elevated blood pressure (SBP 120-129 mmHg and DBP < 80 mmHg); HT1=Hypertension, stage 1 (SBP 130-139 mmHg or DBP 80-89 mmHg); HT2= Hypertension, stage 2 (SBP≥140 mmHg or DBP ≥ 90 mmHg). | | | | | | | |

| **Table S2. Number of subjects according to sex and HT phenotype.** | | | | | | | | |
| --- | --- | --- | --- | --- | --- | --- | --- | --- |
|  | ***MALES*** | | | | ***FEMALES*** | | | |
| ***Category*** | ***ISH (%)*** | ***IDH (%)*** | ***SDH (%)*** | ***Tot*** | ***ISH (%)*** | ***IDH (%)*** | ***SDH (%)*** | ***Tot*** |
| **HT1** | *182 (40)* | *150 (33)* | *125 (27)* | 457 | *17 (7)* | *182 (77)* | *37 (16)* | 236 |
| **HT2** | *52 (15)* | *12 (3)* | *291 (82)* | 355 | *4 (3)* | *8 (5)* | *132 (92)* | 144 |
| NBP= Normal blood pressure (SBP <120 mmHg and DBP < 80 mmHg); EBP= Elevated blood pressure (SBP 120-129 mmHg and DBP < 80 mmHg); HT1=Hypertension, stage 1(SBP 130-139 mmHg or DBP 80-89 mmHg); HT2= Hypertension, stage 2 (SBP≥140 mmHg or DBP ≥ 90 mmHg). ISH=Isolated systolic blood pressure (SBP≥140 mmHg or DBP < 90 mmHg). IDH=Isolated diastolic/predominantly diastolic hypertension (DBP ≥ 90 mmHg and SBP < 140 mmHg). SDH=Systolic-diastolic hypertension (SBP≥140 mmHg and DBP ≥ 90 mmHg). | | | | | | | | |

| **Table S3. Lifestyle characteristics in males** | | | | |  |
| --- | --- | --- | --- | --- | --- |
| ***Parameter*** | ***Normal*** | ***Elevated*** | ***Hypertension, stage 1*** | ***Hypertension, stage 2*** | ***P*** |
|  | ***N=***409  (26%) | ***N=***343  (22%) | ***N=***457  (29%) | ***N=***355  (23%) |  |
| Alcohol (units/week) | 10.70±13.07 | 12.50±12.73 | 14.43±16.05^*^ | 13.61±13.77^*^ | 0.002 |
| Smoking (%) | 9 | 12 | 12 | 16 | 0.2 |
| Exercise (%) | 85 | 86 | 88 | 82 | 0.06 |
| Sodium (mmol/l) | 139.73±12.25 | 140.75±2.67 | 139.78±12.17 | 140.81±2.40 | 0.4 |
| Potassium (mmol/l) | 4.30±0.40 | 4.32±0.37 | 4.32±0.41 | 4.21±0.43 | 0.2 |
| Urea (mmol/l) | 4.94±1.29 | 4.88±1.25 | 4.90±1.34 | 4.84±1.23 | 0.9 |
| Creatinine (μmol/l) | 83.15±12.62 | 86.20±34.73 | 85.46±15.12 | 87.41±12.79 | 0.07 |
| eGFR (ml/min/1.73m^2^) | 114±16 | 113±18 | 111±20 | 107±16^*ŦƗ^ | <0.001 |
| Normal blood pressure= SBP <120 mmHg and DBP < 80 mmHg; Elevated blood pressure=SBP 120-129 mmHg and DBP < 80 mmHg; Hypertension, stage 1=SBP 130-139 mmHg or DBP 80-89 mmHg; Hypertension, stage 2=SBP≥140 mmHg or DBP ≥ 90 mmHg. eGFR=estimated glomerular filtration rate using the Chronic Kidney Disease Epidemiology Collaboration (CKD-EPI) equation^1^. ^*^ P <0.05 versus normal blood pressure. ^Ŧ^ P <0.05 versus elevated blood pressure. ^Ɨ^ P <0.05 versus hypertension, stage 1.   \| **Table S4. Lifestyle characteristics in females** \| \| \| \| \|  \| \| --- \| --- \| --- \| --- \| --- \| --- \| \| ***Parameter*** \| ***Normal*** \| ***Elevated*** \| ***Hypertension, stage 1*** \| ***Hypertension, stage 2*** \| ***P*** \| \|  \| ***N=***1080  (68%) \| ***N=***121  (8%) \| N=236  (15%) \| N=144  (9%) \|  \| \| Alcohol (units/week) \| 6.12±6.19 \| 7.73±6.34 \| 7.02±8.10 \| 5.87±6.07 \| 0.03 \| \| Smoking (%) \| 8 \| 9 \| 11 \| 11 \| 0.2 \| \| Exercise (%) \| 82 \| 83 \| 84 \| 75 \| 0.1 \| \| Hormonal contraception (%) \| 28 \| 34 \| 31 \| 29 \| 0.4 \| \| Sodium (mmol/l) \| 139.74±7.70 \| 140.65±2.29 \| 140.01±2.26 \| 140.05±2.20 \| 0.6 \| \| Potassium (mmol/l) \| 4.28±0.46 \| 4.25±0.35 \| 4.28±0.45 \| 4.18±0.33 \| 0.2 \| \| Urea (mmol/l) \| 4.21±1.11 \| 4.08±1.19 \| 4.10±1.08 \| 4.22±1.29 \| 0.2 \| \| Creatinine (μmol/l) \| 69.65±13.25 \| 74.23±37.73 \| 69.10±12.80 \| 67.87±10.22 \| 0.6 \| \| eGFR (ml/min/1.73m^2^) \| 108±20 \| 105±18 \| 108±19 \| 107±15 \| 0.4 \| \| Normal blood pressure= SBP <120 mmHg and DBP < 80 mmHg; Elevated blood pressure=SBP 120-129 mmHg and DBP < 80 mmHg; Hypertension, stage 1=SBP 130-139 mmHg or DBP 80-89 mmHg; Hypertension, stage 2=SBP≥140 mmHg or DBP ≥ 90 mmHg. eGFR=estimated glomerular filtration rate using the Chronic Kidney Disease Epidemiology Collaboration (CKD-EPI) equation^1^.^*^ P <0.05 versus normal blood pressure. ^Ŧ^ P <0.05 versus elevated blood pressure. ^Ɨ^ P <0.05 versus hypertension, stage 1. \| \| \| \| \| \| | | | | | |

| **Table S5. Heart Rate Variability characteristics in a subgroup of 465 males** | | | | |  |
| --- | --- | --- | --- | --- | --- |
| ***Parameter*** | ***Normal*** | ***Elevated*** | ***Hypertension, stage 1*** | ***Hypertension, stage 2*** | ***P*** |
|  | ***N=***132  (28%) | ***N=***106  (23%) | ***N=***146  (32%) | ***N=***81  (17%) |  |
| NN50 count | 278±181 | 279±182 | 222±180 | 214±186 | 0.009 |
| pNN50 (%) | 38±20 | 37±20 | 32±20^*^ | 27±21^*Ŧ^ | 0.001 |
| RMSSDD (ms) | 78.59±43.55 | 72.26±43.55 | 67.33±43.03 | 59.57±44.64^*^ | 0.02 |
| Triangular index | 566.83±167.83 | 534.65±168.70 | 520.19±166.70 | 499.67±172.99^*^ | 0.03 |
| SDANN (ms) | 19.28±16.72 | 17.93±16.80 | 18.57±16.61 | 17.69±17.24 | 0.9 |
| SDNN (ms) | 84.23±38.74 | 82.57±38.95 | 70.35±38.48^*^ | 67.91±39.95^*^ | 0.003 |
| LF power normalized (n.u.) | 54.27±16.22 | 55.60±16.23 | 55.79±16.12 | 56.59±16.68 | 0.8 |
| HF power normalized (n.u.) | 45.76±16.20 | 44.43±16.28 | 44.24±16.09 | 43.44±16.66 | 0.8 |
| LF:HF | 1.65±1.76 | 1.78±1.77 | 1.73±1.75 | 2.17±1.81 | 0.2 |
| Total power (ms^2^) | 6101.77±5078.88 | 5112.28±5105.38 | 4596.23±5045.45 | 3894.23±5226.12^*^ | 0.02 |
| All data are adjusted for age and ethnicity. Data are means±SD. Normal blood pressure= SBP <120 mmHg and DBP < 80 mmHg; Elevated blood pressure=SBP 120-129 mmHg and DBP < 80 mmHg; Hypertension, stage 1=SBP 130-139 mmHg or DBP 80-89 mmHg; Hypertension, stage 2=SBP≥140 mmHg or DBP ≥ 90 mmHg. NN50count=number of pairs of adjacent NN intervals differing by more than 50 ms in the entire recording. pNN50=NN50 count divided by the total number of all NN intervals. RMSSDD=the square root of the mean of the sum of the squares of differences between adjacent NN intervals. Triangular index= integral of the density of the RR interval histogram divided by its height. SDANN= Standard deviation of the average NN intervals for each 5 min segment of the entire recording. SDNN= Standard deviation of all NN intervals. LF power normalized= relative power of the low-frequency band (0.04–0.15 Hz) in normal units. HF power normalized= relative power of the high-frequency band (0.15–0.4 Hz) in normal units. LF:HF ratio= ratio of LF-to-HF power. Total power=variance of all NN intervals. ^*^ P <0.05 versus normal blood pressure. ^Ŧ^ P <0.05 versus elevated blood pressure. ^Ɨ^ P <0.05 versus hypertension, stage 1. | | | | | |
| **Table S6. Heart Rate Variability characteristics in a subgroup of 496 females** | | | | |  |
| ***Parameter*** | ***Normal*** | ***Elevated*** | ***Hypertension, stage 1*** | ***Hypertension, stage 2*** | ***P*** |
|  | ***N=***356  (72%) | ***N=***26  (5%) | ***N=***73  (15%) | ***N=***41  (8%) |  |
| NN50 count | 292±192 | 330±190 | 271±192 | 232±198 | 0.2 |
| pNN50 (%) | 39±22 | 42±22 | 34±22 | 27±23^*^ | 0.007 |
| RMSSDD (ms) | 77.04±43.63 | 85.49±43.06 | 66.16±43.43 | 60.24±45.18 | 0.03 |
| Triangular index | 512.06±169.72 | 545.46±167.49 | 480.88±168.90 | 450.86±175.72 | 0.08 |
| SDANN (ms) | 16.44±18.91 | 19.59±18.67 | 17.36±18.85 | 17.79±19.50 | 0.9 |
| SDNN (ms) | 70.29±37.66 | 88.78±37.18 | 67.90±37.53 | 59.49±38.85^Ŧ^ | 0.03 |
| LF power normalized (n.u.) | 46.26±17.66 | 45.33±17.45 | 47.23±17.58 | 49.47±18.29 | 0.7 |
| HF power normalized (n.u.) | 53.74±17.66 | 54.67±17.45 | 52.77±17.58 | 50.53±18.29 | 0.7 |
| LF:HF | 1.20±1.30 | 1.07±1.28 | 1.29±1.30 | 1.37±1.34 | 0.8 |
| Total power (ms^2^) | 5154.11±5628.95 | 6118.83±5560.17 | 4431.26±5603.86 | 4495.02±5830.13 | 0.5 |
| All data are adjusted for age and ethnicity. Data are means±SD. Normal blood pressure= SBP <120 mmHg and DBP < 80 mmHg; Elevated blood pressure=SBP 120-129 mmHg and DBP < 80 mmHg; Hypertension, stage 1=SBP 130-139 mmHg or DBP 80-89 mmHg; Hypertension, stage 2=SBP≥140 mmHg or DBP ≥ 90 mmHg. NN50count=number of pairs of adjacent NN intervals differing by more than 50 ms in the entire recording. pNN50=NN50 count divided by the total number of all NN intervals. RMSSDD=the square root of the mean of the sum of the squares of differences between adjacent NN intervals. Triangular index= integral of the density of the RR interval histogram divided by its height. SDANN= Standard deviation of the average NN intervals for each 5 min segment of the entire recording. SDNN= Standard deviation of all NN intervals. LF power normalized= relative power of the low-frequency band (0.04–0.15 Hz) in normal units. HF power normalized= relative power of the high-frequency band (0.15–0.4 Hz) in normal units. LF:HF ratio= ratio of LF-to-HF power. Total power=variance of all NN intervals. ^*^ P <0.05 versus normal blood pressure. ^Ŧ^ P <0.05 versus elevated blood pressure. ^Ɨ^ P <0.05 versus hypertension, stage 1.   \|  \| \| --- \| | | | | | |
